# The Susceptibility of *Pseudomonas aeruginosa* Strains from Cystic Fibrosis Patients to Bacteriophages

**DOI:** 10.1371/journal.pone.0060575

**Published:** 2013-04-24

**Authors:** Christiane Essoh, Yann Blouin, Guillaume Loukou, Arsher Cablanmian, Serge Lathro, Elizabeth Kutter, Hoang Vu Thien, Gilles Vergnaud, Christine Pourcel

**Affiliations:** 1 Univ Paris-Sud, Institut de Génétique et Microbiologie, UMR 8621, Orsay, France; 2 CNRS, Orsay, France; 3 Laboratoire National de Santé Publique, Abidjan, Côte d'Ivoire; 4 The Evergreen State College, Olympia, Washington, United States of America; 5 Hôpital Armand Trousseau, Assistance Publique-Hôpitaux de Paris (APHP), Bactériologie, Paris, France; 6 DGA/MRIS- Mission pour la Recherche et l'Innovation Scientifique, Bagneux, France; The Scripps Research Institute and Sorrento Therapeutics, Inc., United States of America

## Abstract

Phage therapy may become a complement to antibiotics in the treatment of chronic *Pseudomonas aeruginosa* infection. To design efficient therapeutic cocktails, the genetic diversity of the species and the spectrum of susceptibility to bacteriophages must be investigated. Bacterial strains showing high levels of phage resistance need to be identified in order to decipher the underlying mechanisms. Here we have selected genetically diverse *P. aeruginosa* strains from cystic fibrosis patients and tested their susceptibility to a large collection of phages. Based on plaque morphology and restriction profiles, six different phages were purified from “pyophage”, a commercial cocktail directed against five different bacterial species, including *P. aeruginosa*. Characterization of these phages by electron microscopy and sequencing of genome fragments showed that they belong to 4 different genera. Among 47 *P. aeruginosa* strains, 13 were not lysed by any of the isolated phages individually or by pyophage. We isolated two new phages that could lyse some of these strains, and their genomes were sequenced. The presence/absence of a CRISPR-Cas system (Clustered Regularly Interspaced Short Palindromic Repeats and Crisper associated genes) was investigated to evaluate the role of the system in phage resistance. Altogether, the results show that some *P. aeruginosa* strains cannot support the growth of any of the tested phages belonging to 5 different genera, and suggest that the CRISPR-Cas system is not a major defence mechanism against these lytic phages.

## Introduction

*Pseudomonas aeruginosa* is a highly heterogeneous species whose members show various levels of pathogenicity towards plants and animals [1], [2]. It is widely distributed in the environment and is a normal human commensal. *P. aeruginosa* is naturally resistant to many drugs and its capacity to form biofilms makes it very difficult to eradicate, particularly in chronically infected cystic fibrosis (CF) patients [3], [4].

Due to the recent increase in multidrug resistant bacteria, phage therapy is being considered as a therapeutic alternative to antibiotics [5], [6]. It has been used since soon after the 1917 discovery of bacteriophages by Felix d'Herelle at the Pasteur Institute in Paris, mostly in countries of Eastern Europe. Although controlled studies were seldom available, its efficacy has been clearly demonstrated in numerous cases [7], [8]. Phages have the capacity to reach bacteria trapped inside biofilms such as those that form in the lung of cystic fibrosis patients [9], [10]. Therapeutic cocktails such as the “pyophage” formulation widely used in Georgia to treat purulent skin, wound and lung infections, contain many different phages against each of their target pathogens (which are *staphylococcus*, *streptococcus*, *proteus*, *Escherichia coli* and *P. aeruginosa*, for pyophage). Each set must be modified twice a year to retain its ability to lyse a large proportion of the target species [5], [11]. *P. aeruginosa* bacteriophages are numerous, and current knowledge of their diversity shows that they are distributed in at least 7 genera of purely lytic phages (T7-like, ΦKMV-like, LUZ24-like, N4-like, PB1-like, ΦKZ-like, JG004-like) in addition to a similar number of temperate genera [12], [13]. Within each genus, phages with a variety of different host spectra are observed, in part at least reflecting differences in their tail-associated adhesins [12], [14], [15]. Yet the combination of phages used to treat different types of infections remain empirical and usually target not more than 85% of the isolates necessitating the development of a “Sur-mesure” strategy [13]. For this strategy, at least one virulent phage for a patient's given otherwise recalcitrant bacterial strain is identified from a collection, or even *de novo* isolated when none is available, and a therapeutical preparation is freshly made. A major challenge is the regular emergence of bacteria resistant to any given phage, which necessitates the ongoing isolation of new phages or variants of phages targeting different hosts or host receptors.

The mechanisms of bacterial resistance to phages are diverse, driving co-evolution of bacteria and bacteriophages [16], [17], [18], [19]. They involve not only the inhibition of the adsorption of phages on the bacteria but also the injection of DNA, the degradation of phage DNA by restriction enzymes and the recently-identified bacterial immunity system called CRISPR-Cas [17], [20]. CRISPRs (which stand for Clustered regularly interspaced short palindromic repeats) store copies of fragments of foreign DNA called spacers which, together with the Cas proteins, are used to target incoming DNA, leading to their inactivation [21], [22], [23]. In *P. aeruginosa* recent experiments demonstrated a CRISPR-Cas mediated interference against specific temperate phages [24]. The majority of clinical strains of *P. aeruginosa* harbour prophages and it has been suggested that they may play a role in mucoid conversion in CF patients [25]. Interestingly it was shown that some phages belonging to the Mu-like genus carry genes that inactivate the CRISPR-Cas system, illustrating the complex arm-race between phages and bacteria [26].

In past years, we have investigated the diversity of over 600 *P. aeruginosa* isolates using a Multiple Variable Number of Tandem Repeats (VNTR) assay (MLVA) [27], [28]. Many clusters were found, some of them containing more than 10 non-epidemiologically related isolates recovered in multiple countries and representing clonal complexes. Indeed clones are seemingly emerging in relation to antibiotic resistance or in relation to certain diseases such as CF, in a population that is considered as panmictic [27], [29], [30]. We recently showed that many multidrug resistant strains belonging to international clones could be lysed *in vitro* by bacteriophages [31].

Here, we sought to investigate the spectrum of phage-susceptibility in a selection of *P. aeruginosa* strains representative of the species diversity in CF patients, and to characterize the genera and other properties of phages that are particularly successful clinically, as seen in the Republic of Georgia. To look for resistance mechanisms other than lack of relevant surface receptors, we examined the CRISPR-Cas system pattern and showed that it does not play a primary role in resistance to these various lytic phages.

## Materials and Methods

### Ethics statement

The present project is in compliance with the Helsinki Declaration (Ethical Principles for Medical Research Involving Human Subjects). Strains were collected from sputum as part of the patients' usual care, without any additional sampling. The ethic committee “Comité Consultatif pour la Protection des Personnes dans la Recherche Biomédicale (CCPPRB) Ile-De-France” was consulted, specifically approved this study and declared that patient informed consent was not needed.

### Strains

Clinical *P. aeruginosa* strains were isolated from CF patients in the context of longitudinal surveys [27], [28]. The letter C refers to CF, the first digit corresponds to a center and the second digit to the strain index in this center. They were genotyped by MLVA as described previously [27] and their characteristics will be reported in more details (Llanes et al. JAC 2013 in press). Forty seven strains were selected for phage isolation and 18 additional ones were used for CRISPR-Cas investigation. Strains UCBPP-PA14 and PAO1 were purchased from the "Collection de l'Institut Pasteur" (CIP, Paris, France) and C50 was a gift of U. Römling (Karolinska Institutet, Sweden).

### Bacteriophages

The pyophage preparation was obtained from the Eliava Institute (Tbilisi, Georgia). It is a mixture of phages directed against five bacterial species that predominate in purulent infections, including *P. aeruginosa*, and is used for phage therapy [5], [11].

### Culture of bacteria for phages isolation

One colony of *P. aeruginosa* was added to 20 mL Luria Broth (LB) and incubated overnight at 37°C, the culture was centrifuged at 2500 g for 10 min and the bacteria were concentrated 10 times into saline magnesium (SM) buffer (50 mM Tris-HCL pH 7.5, 100 mM NaCl, 8.1 mM MgSO4, 0.01% gelatin) (density of approximately 15 OD_600 nm_).

### Isolation of phages from pyophage

Several dilutions of pyophage were prepared in SM buffer and 10 µL of each dilution were incubated for 15 min at room temperature with 50 µL bacterial suspension before adding 4 mL soft agar (0.7%) LB medium and pouring immediately onto LB agar plates. After overnight incubation at 37°C, a single plaque was picked and purified by several successive platings on host bacteria. Purified phages were amplified on agar plates by infecting 10^8^ bacteria with 10^6^ phages (MOI = 0.01) and incubating at 37°C for 8 hours. A double agar layer plaque assay was used to titrate phage suspensions with10 µL each of different phage dilutions.

### Prophage induction

To induce prophages, 20 mL of an exponential bacterial culture (0.6 OD_600 nm_) was centrifuged and resuspended in 5 mL of 100 mM MgSO_4_. Bacterial cells were transferred onto a Petri dish and placed under a germicide UV light for 30 s before adding 5 mL of fresh LB medium. Irradiated bacteria were then cultured until the absorbance at 600 nm dropped and were pelleted by centrifugation at 2500 g for 10 min at 4°C. The pH of the supernatant was neutralized with 0.1N NaOH and bacteriophage presence was tested by spotting 10 µL onto at least four bacterial strains from the panel of 47. When no lysis was observed the assay was repeated with two more strains.

### Isolation of new phages

New bacteriophages were isolated from sewage samples after overnight enrichment on several bacterial strains. For this, the sewage sample was clarified by centrifugation at 4000 rpm for 20 min and filtered through 45 µm pore size membranes (Sarstedt, Marnay, France). Then, 5 mL of filtrate were incubated overnight with selected bacterial strains (200 µL of a tenfold concentration of an overnight culture) and 10 mL of LB medium at 37°C. About 100 µL of chloroform were added to the culture and bacteria debris were removed by centrifugation at 2500 g for 10 min. Ten microliters of the supernatant were spotted onto different bacterial strains including the strain used for phage enrichment. Phages were recovered from lysis zones and purified by two cycles of plating on the enrichment bacterial strain. Following the proposal by Kropinski et al. [32], the full name of a bacteriophage will include vB for virus of bacteria, and indication of the host species and the virus family. For example the first phage isolated on *P. aeruginosa* strain C1-14 in Orsay will be called vB_Pae_M-C1-14_Or01_. "M" indicates that the phage is a myovirus.

### Phage host range

In order to determine phage host range, 10 µL of concentrated phages (titer≥10^9^ “plaque forming units” PFU per mL) were spotted on stationary growing bacteria and plaque assays were used to check the sensitivity of bacteria. This assay was repeated three times. For those testing positive, their efficiency of plating was determined by spotting 5 µL of phages at ten-fold serial dilutions (10^−1^–10^−8^) onto the bacterial lawn. Similarly, phages cocktails prepared by mixing 10^7^ PFU of each phage, were tested using serial dilutions.

### Electron microscopy

Phages were amplified by culturing with selected bacterial strains on fresh LB agar plates for 8 hours at 37°C, and the soft agar was recovered in 4 mL of SM buffer and a few drops of chloroform. After centrifugation at 2500 g for 10 min, the supernatant was filtrated through 0.45 µm pore size membranes, and the phages were pelleted by ultracentrifugation (Beckman Coulter SW41 rotor) at 260,000×g for 2 hours at 4°C. Phages were purified by two washings with 100 mM ammonium acetate pH 7 and the pellet was finally resuspended in 100 µL SM buffer. Then 3 µL of concentrated bacteriophages were spotted on carbon-coated grids and were allowed to adsorb for 5 min. Phages were stained by adding a drop of 2% potassium phosphotungstate (pH 7) for 30sec, and excess sample was removed by carefully touching the side of the grid with filter paper. The grid was then visualized using an EM208S transmission electron microscope (FEI, Eindhoven, The Netherlands) operating at 80 kV.

### DNA preparation and restriction enzyme digestion

For rapid DNA purification, phages were amplified by culturing on fresh LB agar plates for 8 hours at 37°C. Then 5 mL of a 10 mM Tris PH 7.5, 10 mM MgSO_4_ solution were added to the plate followed by overnight incubation at 4°C. The buffer was transferred to a 50 mL tube and bacteria debris were pelleted by centrifugation at 2500 g for 10 min at 4°C. A mixture of 0.2 mL 2 M Tris pH 7.5, 0.4 mL 0.5M EDTA, 0.2 mL 10% SDS and 10 µL diethylpyrocarbonate was added to 4 mL of supernatant. Following incubation at 65°C for 30 min, the tube was cooled on ice and 1 mL 5M KOH was added. After 1 hour incubation in ice, centrifugation was performed at 25000 g for 20 min at 4°C. The DNA contained in the supernatant was precipitated with 2 volumes of absolute ethanol, pelleted by centrifugation, washed twice with 70% ethanol, dried and dissolved in 0.4 mL 10 mM Tris pH 7.5, 1 mM EDTA. For restriction enzyme analysis, 10 µL bacteriophage DNA was digested according to the manufacturer's recommendations and analysed on a 0.8% agarose gel in 0.5× TB buffer. In some cases, digestion was carried out overnight to ensure total digestion. The restriction enzymes were *Eco*RI, *Hind*III, *Bss*HII, *Bgl*II, *Sau*3A, *Alu*I, *Hae*II.

### Phage whole genome sequencing

Phage DNA was prepared using the rapid protocol described above and the quality was checked by restriction enzyme digestion, followed by gel analysis. Then 5 µg were sent to the CNRS sequencing facility in Gif sur Yvette (IMAGIF) for preparation of a library and sequencing on the Illumina platform (Illumina Genome Analyzer IIx). Paired-end 75 bp reads were produced, from mean insert length around 300 bp. The BioNumerics tools (Applied Maths, Sint-Martens-Latem, Belgium) were used for assembly and comparison of genome sequences. For the *de novo* assembly, two algorithms were applied to produce contigs, namely Velvet [33] and Ray [34]. Both are implemented in the 6.6 version of the BioNumerics software, in the "Power Assembler" module. A single contig was obtained without any gap and the distribution of reads along this contig was analysed. After alignment with published sequences large regions of deletion were verified by PCR amplification using primers selected in flanking sequences. Annotated sequences were deposited at EMBL-EBI under accession number HE983845 for phage vB_PaeM_C2-10_Ab01, and HE983844 for phage vB_PaeP_C1-14_Or01. Additional information is available at the web site http://bacteriophages.igmors.u-psud.fr.

### Amplification and sequencing of CRISPR and of *cas* genes

Two CRISPR-Cas systems have been observed in sequenced genomes of *P. aeruginosa*. The type I–F (*Yersinia*) system or CASS3 subtype [35] found in strain PA14 includes two CRISPRs with 28 bp repeats. They are referred to as NC_6483_2 or CRISPR1 with repeat TTTCTTAGCTGCCTATACGGCAGTGAAC and NC_006483_3 or CRISPR2 with repeat TTTCTTAGCTGCCTACACGGCAGTGAAC [36]. They are associated with a set of *cas* genes, from among which *cas*1 was selected for amplification. The primers used for *cas*1 and CRISPR PCR amplification are shown on Table 1. In the unpublished draft genome sequence of strain 2192 [37] (NZ_AAKW00000000, available from the Broad Institute web site www.broad.mit.edu/annotation/genome/pseudomonas_group/MultiHome.html) the type I–F CRISPR-Cas system is present together with another system composed of three CRISPRs with a 29 bp repeat and another set of *cas* genes. This system is called type I–E (*Escherichia*) or CASS2 [38]. Cse3_2192 was amplified using primers Pseudomonas cse3 For and Pseudomonas cse3 Rev described by Cady et al. [39].

**Table 1:** Primers used for PCR amplification in the present study.

Primers	Sequence 5′ to 3′	Reference
CRISPRPaer-F	CTTGACGACCTATGTGGCAG	This study
CRISPRPaer-R	GTCGCTGCAAAAAGAGGGTT	This study
CRISPR2Paer-F	TTTTCGTCTGTGTGAGGAGC	This study
CRISPR2Paer-R	AGCAAGTTACGAGACCTCGA	This study
CRISPR3Paer-F	ATTTCCAGGAGCGGCGCGAG	This study
CRISPR3Paer-R	GATCACGCCACTGGTCGTAG	This study
PaerCas1_F	GACATTTCTCCCAGCGAACTGA	This study
PaerCas1_R	CTTCTTCGGTCAGTAGATGCTC	This study
Pseudomonas *cse3* For	ATGTACCTGACCAGACTGACCCTTGATCCTCGCAGCG	[39]
Pseudomonas *cse3* Rev	GGCTCAGCAGGCCACAGCCGAAAGCCTTG	[39]

PCR products were sequenced by Eurofins MWG biotech after purification using the DNeasy Blood & Tissue kit (QIAGEN). To classify the spacers in CRISPRs, the CRISPRtionary tool at the CRISPRcompar web site was used (http://crispr.u-psud.fr/CRISPRcompar/) [40]. A dictionary of spacers was produced for CRISPR1 in the orientation based on repeat sequence TTTCTTAGCTGCCTATACGGCAGTGAAC such as described by Cady et al. [39].

## Results

### Purification of bacteriophages from pyophage on selected *P. aeruginosa* strains

**Figure 1:**
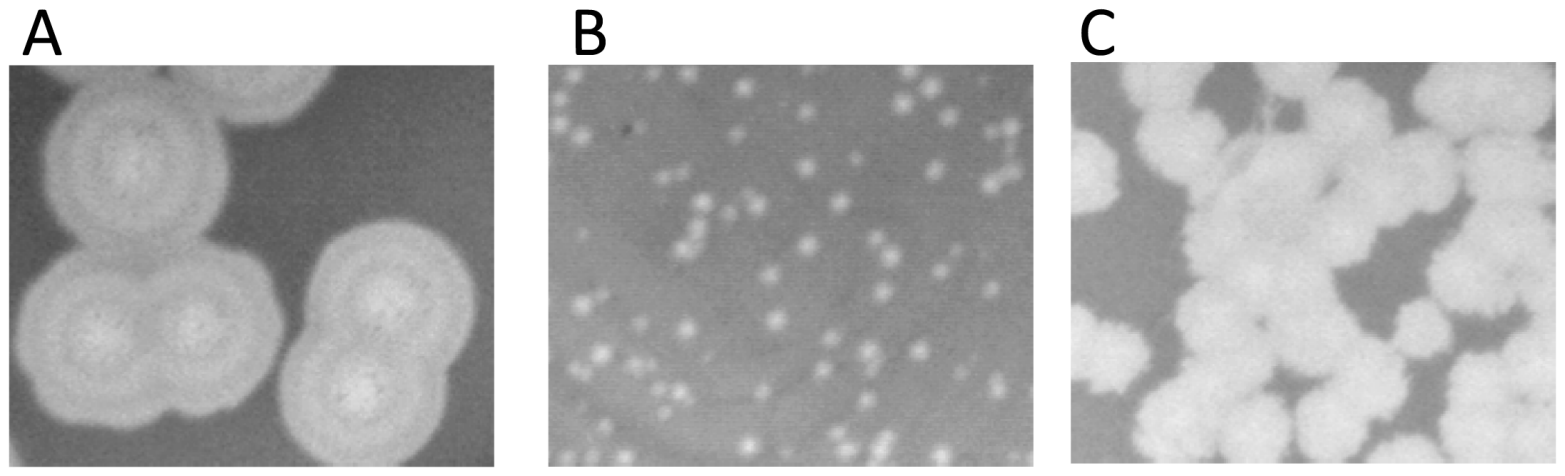
Morphology of plaques produced by three different phages of the pyophage A) P1-15_pyo_ on PAO1, B) P1-14_pyo_ on C1-14, C) PTr60_pyo_ on Tr60.

The complex commercial Eliava “pyophage” formulation has a very broad host range, built up over the years, but the details of its composition are unknown. To isolate bacteriophages from pyophage and to test their host ranges, 47 *P. aeruginosa* strains were selected from among 325 strains from French CF patients, on the basis of their distribution into different MLVA clusters (Fig. S1). Two to three members each of 14 MLVA clusters were chosen in order to check whether a similar pattern of phage susceptibility would be observed within each cluster. In addition three reference laboratory strains, PA14, PAO1 and C50, were also used. In about 90% of the strains, the presence of prophages or pyocins could be demonstrated following UV activation and spot testing onto 4 to 6 different strains among the panel of 47 (data not shown). To isolate phages, the pyophage preparation was titered at 10^−1^, 10^−2^, 10^−3^ and 10^−4^ dilution on a subgroup of 14 CF strains, distributed into the different MLVA clusters, and on the reference strains PAO1 and C50. On C50, C3-16, C7-12, C7-25, C8-20 and C9-6 no plaques were seen whereas plaques of different sizes and morphologies were observed on the other strains. A total of 10 plaques were recovered from 10 different bacterial strains (C1-14, C1-15, C2-10, C3-20, C5-13, C8-5, C8-13, C8-17, PAO1, Tr60) and purified by three successive platings on the same strain. Different plaque sizes and shapes were observed and were characteristic for a phage on a given strain (three examples are shown on Fig. 1). All phages produced clear plaques after two rounds of infection on a strain. On PAO1, four phages produced very large plaques surrounded by a halo, which kept enlarging with time, suggesting the presence of a strong depolymerase activity (Fig.1A).

**Figure 2:**
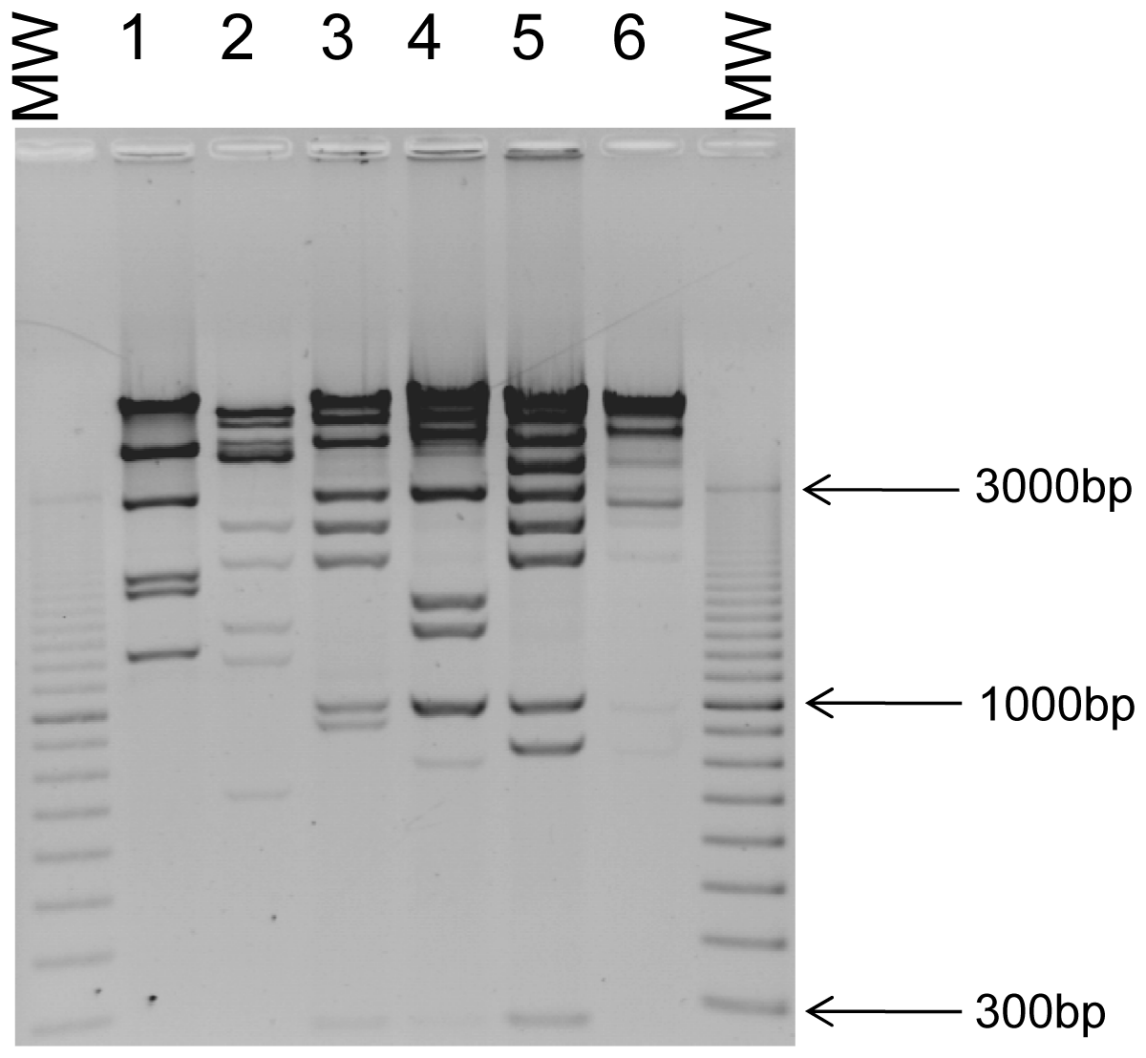
Restriction analysis of phage DNA. The DNA of each of six phages from pyophage was digested by *Hind*III and electrophorezed on a 0.8% agarose gel. From 1 to 6: P1-15_pyo_, P8-13_pyo_, P2-10_pyo_, P3-20_pyo_, PTr60_pyo_, P1-14_pyo_. MW is a size marker. On the side are indicated the sizes of three DNA fragments. The DNA of P1-14_pyo_ is not totally digested.

**Figure 3:**
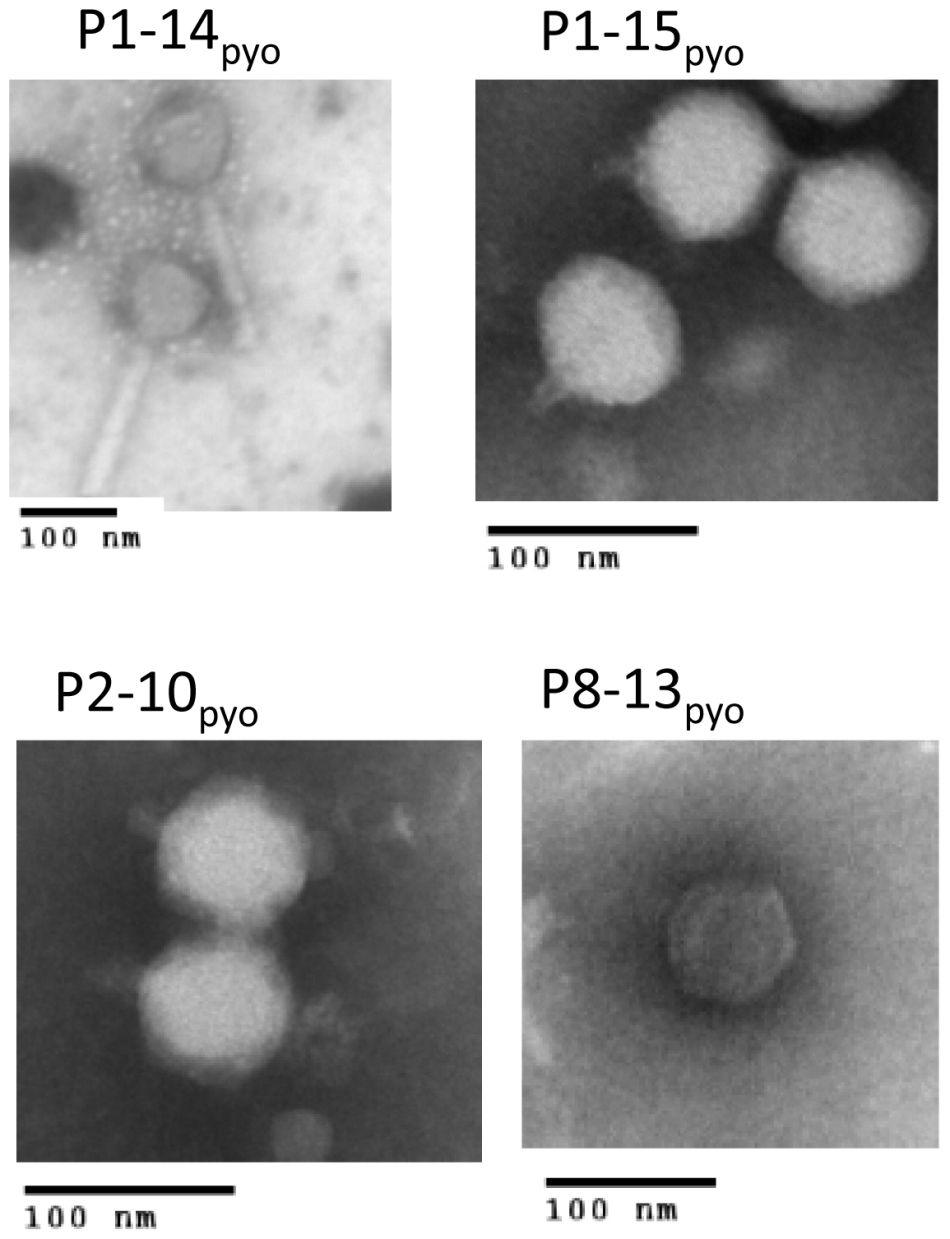
Electron microscopy observation of four phages from pyophage: P8-13_pyo_, P1-14_pyo_, P1-15_pyo_, P2-10_pyo_.

The restriction profiles of phage genomic DNA were investigated using several enzymes. *EcoR*I, *Hind*III, *Sau3*A and *Alu*I were able to restrict all of the samples, although full digestion of P1-14_pyo_ was not observed with *EcoR*I and *Hind*III. The four phages producing a large halo had the same profile with different enzymes (data not shown) and only one, P1-15_pyo_, was retained for further analysis. Finally, six phages showing different *Hind* III restriction profiles were selected (Fig. 2). Sequencing of several genomic fragments and evaluation of the genome size by analysis of restriction enzyme patterns, suggested that P1-15_pyo_ is a ΦKMV-like phage, P8-13_pyo_ is an N4-like phage, P2-10_pyo_, P3-20_pyo_ and PTr60_pyo_ are LUZ24-like phages, and P1-14_pyo_ is a PB1-like phage (Fig. S2 and data not shown) [12]. In agreement with these results, electron microscopy analysis showed that phage P1-14_pyo_ with a 72 nm diameter head and a 140–142 nm long tail belonged to the *Myoviridae* family, whereas P1-15_pyo_, P8-13_pyo_ and P2-10_pyo_ with isometric heads of respectively 63–65 nm, 72 nm and 58–60 nm diameter belonged to the *Podoviridae* (Fig. 3). The morphology of P3-20_pyo_ and PTr60_pyo_ was similar to that of podovirus P2-10_pyo_ (data not shown).

### Virulence of the pyophage-isolated phages

We evaluated the host range and virulence of the six pyophage-derived phages toward the full collection of 47 *P. aeruginosa* strains representative of the diversity in CF patients and towards the three reference strains, by spotting a concentrated suspension onto a bacterial lawn. Table 2 shows the bacteriophage virulence profile. No clear lysis zone could be seen even with high phage concentrations on eleven strains (C50, C5-2, C7-6, C7-12, C7-25, C7-26, C8-7, C8-14, C8-15, C8-20, C9-6) and only a turbid zone or trace growth was observed on 3 additional strains (C3-16, C5-13, C9-5) in good agreement with the lack of susceptibility to the pyophage cocktail presented in the previous paragraph. The other strains showed different susceptibilities to the six phages, mostly independent of their MLVA genotypes. In four cases, strains with the same MLVA genotype showed the same or highly similar phage susceptibility (C8-14 and C8-15, C10-3 and C10-4, C7-25 and C7-26, C8-7 and C8-20) but they corresponded to strains from epidemiologically related cases. In other instances, strains with the same MLVA genotype originating from patients in different hospitals displayed different phage susceptibility profiles (C50 and C10-5; C3-13, C5-13 and C10-1; C1-7 and C3-16; Tr60, C7-11 and C8-12).

**Table 2:** Susceptibility of 50 *P. aeruginosa* strains to 6 pyophage-derived phages, and CRISPR content.

	N4-like	Φ-KMV-like	PB1-like	LUZ24-like			type I–F			type I–E
	p8-13_pyo_	p1-15_pyo_	p1-14_pyo_	pTr60_pyo_	p3-20_pyo_	p2-10_pyo_	CRISPR1	CRISPR2	*cas*1	*cas*1
**PAO1** a	C^b^	C	-	-	-	-	-	-	-	-
PA14	-	-	C	-	-	T	+	+	+	-
**C50**	T	-	-	-	-	-	+	+	+	-
**Tr60**	-	C	C	C	C	C	+	+	+	-
C1-1	C	C	T	C	-	-	+	+	+	-
**C1-2**	T	T	T	T	T	T	-	-	-	-
C1-3	T	-	C	C	C	C	-	-	-	-
C1-4	-	-	-	C	C	C	-	-	-	-
C1-7	C	-	C	T	-	T	+	+	+	-
C1-11	-	-	-	C	C	T	+	+	+	-
**C1-14**	C	C	C	C	T	T				
**C1-15**	-	C	-	C	-	-	+	+	+	-
C2-3	T	-	-	C	C	T	+	+	+	-
**C2-10**	-	-	-	C	C	C	-	-	-	-
C3-13	C	T	C	T	-	-	+	+	+	+
C3-14	C	-	-	C	C	C	-	-	-	-
**C3-16**	C	-	-	-	-	-	+	+	+	+
C3-17	-	-	C	C	C	C	-	-	-	+
**C3-20**	T	-	-	C	C	C	-	-	-	-
C4-12	-	T	-	C	C	T	-	-	-	-
C4-17	T	T	-	C	C	C	+	+	+	-
C5-2	-	-	-	-	-	-	+	+	+	-
C5-12	-	-	T	T	T	T	+	+	+	-
**C5-13**	-	T	-	-	-	-	-	-	-	+
C7-6	-	-	-	-	-	-	-	-	-	-
C7-11	-	-	C	C	C	C	+	+	+	-
**C7-12**	-	-	-	-	-	-	-	-	-	-
C7-22	-	-	-	C	C	C	-	-	-	-
**C7-25**	-	-	-	-	-	-	-	-	-	-
C7-26	-	-	-	-	-	-	-	-	-	-
**C8-5**	T	T	-	T	T	-	+	+	+	-
C8-7	-	-	-	T	-	-	-	-	-	-
C8-12	-	-	C	T	T	T	+	+	+	-
**C8-13**	T	-	C	C	C	C	+	+	+	-
C8-14	-	-	-	-	-	-	-	-	-	-
C8-15	-	-	-	-	-	-	-	-	-	-
**C8-17**	-	C	-	C	-	-	+	+	+	-
C8-19	-	-	-	C	T	T	+	+	+	-
**C8-20**	-	-	-	-	-	-	-	-	-	-
C9-2	-	-	-	C	-	-	-	-	-	-
C9-5	-	T	-	T	-	-	-	-	-	-
**C9-6**	-	-	-	-	-	-	-	-	-	-
**C9-11**	C	C	C	C	C	-	+	+	+	-
C9-17	-	-	T	-	T	-	+	+	+	-
C10-1	C	-	-	-	-	-	+	+	+	+
C10-2	C	-	C	C	C	C	+	+	+	-
C10-3	C	-	-	-	-	T	-	-	-	-
C10-4	C	-	-	-	-	T	-	-	-	-
C10-5	C	-	T	T	C	T	+	+	+	-
C10-6	T	-	-	T	C	C	-	-	-	+

a in bold are shown strains used to purify phages from pyophage.

b C means clear lysis zone and T means turbid lysis zone.

### Isolation and whole genome sequencing of two new phages

**Figure 4:**
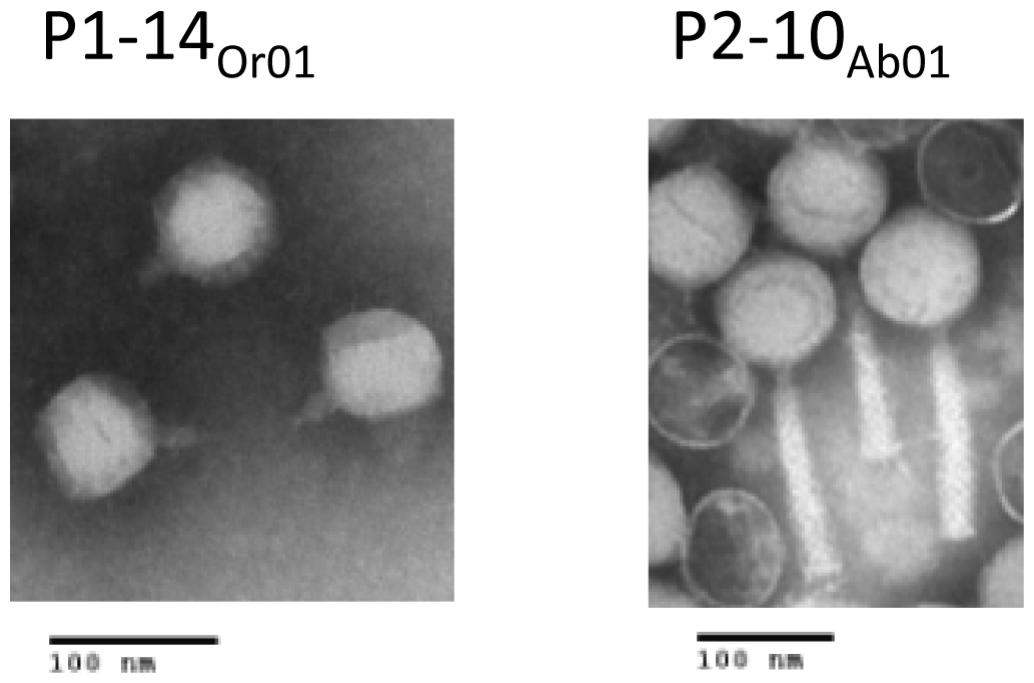
Electron microscopy observation of the new phages P1-14_Or01_ and P2-10_Ab01_.

Different strains that were not susceptible to pyophage were used to enrich for phages in sewage water from Orsay (France) and Abidjan (Ivory Coast) and one from each place was selected for further characterization. One phage isolated in Orsay using C8-15, and thereafter amplified on C1-14, was called “vB_PaeP_C1-14_Or01” abbreviated as P1-14_Or01_. It is a podovirus as shown by EM analysis, with a 60 nm head and a short tail (Fig. 4). Whole-genome sequencing was performed using the Illumina technology and a single contig of 45,469 nucleotides was assembled. Interestingly, when we analysed the reads coverage along this contig we noticed the presence of high peaks with one defined end on one strand (Fig. S3). Two peaks could be assigned to the genome ends (labelled RE and LE Fig S3A) and 8 peaks were distributed along the genome. At the 8 sites we found a short conserved sequence which consensus is GTCATAGTAC (Fig. S3B).

**Figure 5:**
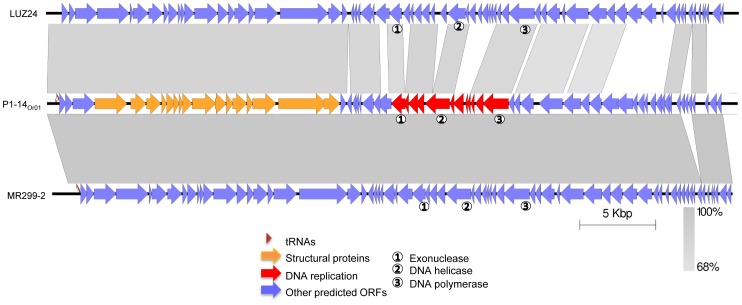
Alignment of P1-14_Or01_, MR199-2 and LUZ24 genomes. Putative open reading frames (ORF) are shown with arrows. In red are shown the tentative cluster of genes encoding structural proteins and in yellow the genes involved in DNA replication. tRNA genes are shown with triangles. Boxes with different shades of grey represent degrees of similarities between genomes.

Upon alignment of the P1-14_Or01_ genome with sequenced genomes present in Genbank, the closest phages were ΦMR299-2 with 97% average nucleotide similarity [10], PaP3 with 95% average similarity [41], and LUZ24 with 90% average similarity [42]. The differences were not evenly distributed but were concentrated into several regions with 84 to 94% identity with the closest phage genome ΦMR299-2. An approximately 3000 bp fragment showed 97% similarity with phage PaP3 and 86% similarity with phage ΦMR299-2. It encompassed 3 hypothetical open reading frames (orf10 to orf13), two of which (orf12 and orf13) encode constituents of the phage particle. Another region of about 660 bp showed 91% identity with ΦMR299-2 and 92% identity with LUZ24 and encompassed orf17 encoding a putative phage particle protein. Finally a 600 bp region with 84% identity with ΦMR299-2 and 96% identity with PaP3 encoded orf25, a putative phage constituent protein. Figure 5 shows the gene organisation of P1-14_Or01_ and a comparison with that of phages ΦMR299-2 and LUZ24. A 182 bp sequence similar to the LUZ24 long terminal repeat sequence could be assembled on one end. Three tRNA genes (tRNA^Asn^, tRNA^Asp^ and tRNA^Pro^) were present in P1-14_Or01_ and phiMR199-2 compared to four in PaP3 and two in LUZ24. Phage ΦMT299-2 is lacking a 734 bp region, as compared to P1-14_Or01_, encompassing three putative genes (orf58 to orf60 in P1-14_Or01_). No genes potentially involved in lysogeny (integrase, recombinase, repressor, or excisionase) were detected in this phage.

**Figure 6:**
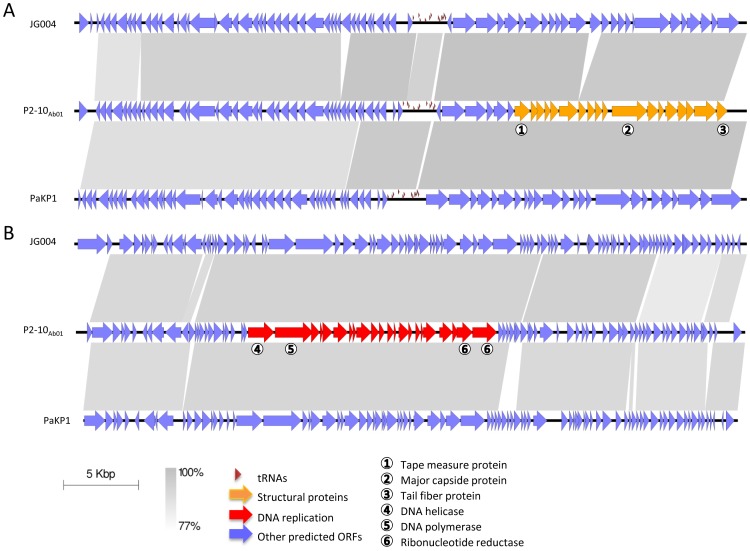
Alignment of P2-10_Ab01_, JG004 and PAKP1 genomes. The genomes are separated into two sections (A and B) of approximately 46 kb. The legend is that of Figure 4.

The second new phage recovered from a sewage water sample from Abidjan by enrichment with pyophage-resistant strain C7-6 produced large clear plaques on this strain, and on C9-5 but with a low efficiency. The highest degree of growth was seen on C2-10 and C9-11. It is a myovirus as shown by EM examination, with a 85 nm diameter head and a 142 nm long tail (Fig. 4). This phage, which was amplified on C2-10, was named “vB_PaeM_C2-10_Ab01” abbreviated as P2-10_Ab01_. Genome sequencing produced a single contig of 92,777 bp showing 93% average sequence similarity with JG004 [43], and 91% average similarity with PaKP1 [44] and PaP1 (HQ832595) which are JG004-like phages. Eleven tRNA genes were found (tRNA^Gln^, tRNA^Arg^, tRNA^Lys^, tRNA^Leu^, tRNA^Ile^, tRNA^Asp^, tRNA^Cys^, tRNA^Pro^, tRNA^Gly^, tRNA^Phe^, tRNA^Glu^) compared to twelve in JG004 and PaKP1 (the same eleven plus tRNA^Asn^). Alignment of P2-10_Ab01_, PaKP1 and JG004 genomes (Fig. 6 A and B) emphasized the mosaic composition of the genomes and showed that the structural genes have the highest degree of conservation. The gene organization was similar, apart from short orfs which were differentially annotated in the three genomes. PJG4_036 and PJG4_070 encode two putative homing endonucleases which were absent from PaKP1 and from P2-10_Ab01_, as confirmed by PCR using flanking primers (data not shown).

The susceptibility of 20 *P. aeruginosa* strains to the two newly isolated phages was investigated by spotting serial dilutions of phages (Table 3). P1-14_Or01_ differs from the three LUZ24-like phages isolated from the pyophage preparation by it virulence for C5-12 and by a low level of growth on C8-15 (Table 2 and Table 3). Interestingly P1-14_Or01_ systematically produced both clear and turbid plaques on C2-10, even after purification of a single plaque, whereas only clear plaques were seen on C1-14, C5-12 and C8-15. P2-10_Ab01_ produced plaques on C7-6 and C9-5 although with approximately 10 times lower efficiency as compared to growth on C2-10 and C9-11. Several strains which did not support growth of the pyophage-derived phages were not susceptible to the new phages (C5-2, C7-12, C7-25, C8-20). Partial 16S RNA gene amplification and sequencing was performed on DNA from these strains confirming that they belonged to the *P. aeruginosa* species.

**Table 3:** Susceptibility of 17 *P. aeruginosa* strains to two newly isolated phages.

	P1-14_Or01_	P2-10_Ab01_
PAO1	t^a^ ++	-
PA14	-	t++
C50	t+	-
Tr60	c+++	t++
C1-14	c+++	c+
C2-10	c+++	c+++
C3-16	t++	t+
C3-20	t++	-
C5-2	-	-
C5-12	c+++	-
C7-6	t+	c+
C7-12	-	-
C7-25	-	-
C8-7	-	t++
C8-14	t+	-
C8-15	c+	-
C8-20	-	-
C9-5	t+	c+
C9-6	-	-
C9-11	t+	c+++

a "t" - turbid plaques; "c" - clear plaques; "-" - no growth; "+" - degree of growth.

### Susceptibility of bacteria to phage cocktails

Three phages mixtures were prepared, respectively composed of the 6 pyophage-derived phages (6 ph cocktail), the two newly isolated phages P1-14_Or_ and P2-10_Ab01_ (2 ph cocktail) and the combination of 8 isolated phages (8 ph cocktail) together representing 5 genera (Table 4). All mixtures contained 10^7^ PFU/mL of each phage. Susceptibility of bacteria toward pyophage and toward 6 ph and 8 ph cocktails were similar except for strains C7-6 and C9-5 which were lysed by P2-10_Ab01_ present in 6 ph and 8 ph. The trace growth seen for C8-15 using P1-14_Or_ alone was not observed in the 2 ph and 8 ph cocktails. The concentration of phages in the pyophage was probably lower than in the other cocktails explaining the lower level of growth on different strains. All the strains that did not support the growth of pyophage were equally not infected by the 6 ph cocktail. Altogether these results demonstrate that the association of several phages does not affect the activity of a given phage.

**Table 4:** Bacteria susceptibility to four phage-cocktails.

	Pyophage	6 ph^a^	2 ph^b^	8 ph^c^
PAO1	c+++	c+++	t++	c+++
PA14	c+++	c+++	-	c+++
Tr60	c+	c+++	c+++	c+++
C50	-	-	-	-
C1-2	c+++	c+++	c+++	c+++
C1-14	c+++	c+++	t+	c+++
C2-10	c+	c+++	c+++	c+++
C3-16	-	-	-	-
C3-20	c+	c+++	t++	c+++
C5-2	-	-	-	-
C5-12	t+	c+++	c+++	c+++
C7-6	-	-	c+	c+
C7-12	-	-	-	-
C7-25	-	-	-	-
C8-5	t+	t+	t+	t+
C8-7	-	-	-	-
C8-14	-	-	-	-
C8-15	-	-	-	-
C8-20	-	-	-	-
C9-2	c+++	c+++	c+++	c+++
C9-5	t+	t+	c+++	c+++
C9-6	-	-	-	-
C9-17	c+++	c+++	c+++	c+++

a 6 ph is a mixture of the 6 pyophage-isolated phages.

b 2 ph is a mixture of phages P1-14_Or01_ and P2-10_Ab01_.

c 8 ph is a combination of 6 ph and 2 ph.

### CRISPR and *cas*1 gene analysis

In order to investigate the potential role of the CRISPR-Cas system in resistance to infection by these phages, we first searched for the presence of the type I–F (*Yersinia*) system in the selected *P. aeruginosa* strains. We used primer sequences localised in the *cas*1 gene because this gene has been shown to be essential for the activity of the CRISPR-Cas system. We also used primers localised on both sides of the CRISPR1 and CRISPR2 loci in PA14 (Fig. S4). In 24 strains, including PA14 and C50, PCRs were positive for all three loci (Table 2). The CRISPRs size varied from one strain to the other. To analyse the DR and spacer diversity, CRISPR1 was sequenced in all these strains and in 18 additional ones from the same collection, resulting in sequences for a total of 264 spacers (Table S1). Common spacers were observed in strains belonging to clusters defined by MLVA (Fig. S5 and S6). A search for sequences homologous to spacers by BLAST in Genbank gave a 100% match for 40% of the spacers, corresponding mostly to temperate phages or prophages but in a few cases to sequences of plasmids, pyocins or pathogenicity islands. A given CRISPR often contained several fragments of a particular phage. In order to compare the CRISPR of our strains with those described in the literature we produced a combined dictionary of spacers found in the study of Cady et al. [39] and the spacers described in the present study (Table S2). Twenty six of our strains possessed spacers that were previously found in other strains.

We also investigated the presence of the type I–E (*Escherichia*) CRISPR-Cas by amplification using primers in the *cas*1 gene. Six strains possessed the type I–E (*Escherichia*), three of which had also the type I–F (*Yersinia*) (Table 2). Interestingly, there was an inverse relationship between the presence of a CRISPR-Cas system and absence of growth of our phages.

## Discussion

### The pyophage preparation

The pyophage formulation is continuously enriched by new phages and variants of these in response to changes in pathogen targets and it is not clear how many species are present [6]. The collection of strains we have used both to purify phages from pyophage and to test their virulence spectrum is representative of the diversity of clinical (CF) *P. aeruginosa* in French hospitals. The most susceptible *P. aeruginosa* strains in this collection were C1-14 and C2-10. A large variety of plaque morphologies could be observed on these strains. The present work allowed the identification of 6 phages showing different restriction enzyme profiles and belonging to four genera [12]. P1-15_pyo_, a ΦKMV-like phage, apparently produces an important depolymerase activity which may be of interest to lyse bacteria inside biofilms [45]. Three phages belonging to the LUZ24-like genus show the largest virulence spectra, with few differences between them, although their restriction profiles were rather different. Other phage species may be present in the pyophage but may have been missed on our collection of strains, because of their lower concentration, inability to target the particular strains being used or pure chance. For example, a JG004-like phage was isolated from pyophage in studies involving a large set of CF strains from Children's Hospital in Seattle, as well as phages from 3 of the genera observed above but with different host ranges (E. Kutter, unpublished). Further work including bulk sequencing will be needed to fully characterize the pyophage cocktail (Mzia Kutateladze and Marina Goderdzishvili, work in progress).

### Isolation and sequencing of new phages

From Orsay sewage water a new phage, P1-14_Or01_, was isolated which could lyse to some extent the pyophage-resistant strain C8-15. Its genome is close to that of phage PAP3 isolated in China [41] and to that of ΦMR299-2 isolated in Ireland [10]. They all belong to the LUZ24-like genus but show substantial variability. PAP3 was reported to be capable of lysogenization (by integration into a tRNA gene rather than by the usual integrase), but we did not find any such evidence for P1-14_Or01_, nor did investigators studying LUZ24 and its other relatives. We observed a very important relative amount of sequencing reads at 8 positions along the genome and detected the consensus sequence GTACTATGAC at each of these positions. This sequence is very close to the consensus sequence 5′-TACTRTGMC-3′ corresponding to single-strand DNA interruptions in phage tf of *P. putida* [46]. Such nicks were never described in *P. aeruginosa* LUZ24-like phages although the site is present in all of the sequenced genomes reported so far. We believe that the DNA preparation method and/or the sequencing technique utilized in the present study is a convenient way to reveal the existence of nicks in phage genomes.

Phage P2-10_Ab01_ isolated from sewage water in Abidjan could lyse two pyophage-resistant strains, C7-6 and C9-5. Its genome is related to those of JG004 and other phages from Poland, France, Japan and Germany, representing a genus we did not detect in pyophage using our strains. Comparison of these phage genomes shows an important degree of mosaicism although the overall arrangement and gene distribution is similar. P2-10_Ab01_ and PAKP1 apparently lack two genes encoding putative homing endonucleases which were observed in phage JG004. JG004 orf36 encodes a 219 amino acids HNH endonuclease which shows similarities with homing endonucleases possessing a C-terminal AP2 DNA binding domain, found in many plants and some bacterial and viral proteins [47]. Genes of this family are present in different bacteriophages including *Salmonella* phage Felix01 [48] and *P. chlororaphis* phiKZ-like phage 201phi2-1 (orf35) [49]. Orf70 encodes a 258 amino acid putative endonuclease with a N-terminal GIY-YIC catalytic domain similar to that of the SegA–E proteins of phage T4 [50] and also present in many bacteriophages. Homing endonucleases can transpose and duplicate themselves and can move via lateral gene transfer [51]. Their abundance in some genomes such as that of *Xanthomonas oryzae* phage Xp10 genome [52] or phage T4 [53] suggests that they play a role in the evolution of phages. They influence the distribution of sequences flanking their insertion site, whether this site is specific or not.

P2-10_Ab01_ and P1-14_Or01_ were able to grow on some strains that were not infected by the pyophage-derived phages. This may be due to the existence of a specific receptor in the case of phage P2-10_Ab01_ which belongs to a new genus. In the case of P1-14_Or01_ belonging to the LUZ24-like genus as three pyophage-derived phages, it might be due to a mutation in the tail binding domain.

New phages are continuously isolated for *P. aeruginosa,* and their capacity to target various clinical strains is tested *in vitro* and in animal models [10], [54], [55]. Our first attempt to obtain phages that could lyse strains that do not support pyophage infection led in part to the isolation of phages which belong to different species or genera. Most new phages belong to already-recognized genera even though they may have new ranges of host specificities. From our observations we suggest that an efficient cocktail of lytic phages for treatment of CF patients should contain phages belonging to different genera such as proposed previously [56]. However it is not clear if the virulence of different phages in a cocktail will add up or if on the contrary different phage types may not be compatible [57]. Our own observations suggest that phages behave similarly alone or in a cocktail. Work done with *E. coli* T4-like phages demonstrates a large variety of host ranges inside a single group [58], [59], and a quite broad variation of host specificity have also been observed between members of some of the genera of *Pseudomonas* phages (E. Kutter, unpublished studies of phages targeting a set of 200 CF strains and phages targeting a set of 100 dog-ear strains). In cocktails containing multiple members of the same genus with different host ranges, there is a substantial possibility for recombination between such phages to generate new host specificities. It is quite probable that this occurs on a regular basis when pyophage is produced and used.

### Mechanisms of resistance and the role of the CRISPR-Cas system

Thirteen out of 47 *P. aeruginosa* strains, including members of clone C frequently isolated from CF patients, were not lysed by the pyophage preparation or by each phage individually. Some were also not susceptible to a much larger collection of phages from different geographical origins and belonging to other genera such as ΦkZ-like phages (V. Krylov personal communication). Loss or significant alteration of receptors or overproduction of exopolysaccharides such as alginates may represent general mechanisms of resistance [19]. The existence of a particular restriction system is also possible. These strains were not genetically related, were not mucoid, and were susceptible to most antibiotics. The lung of CF patients is colonized very early by multiple bacterial species which coexist with bacteriophages inside a biofilm [60]. This might create a favourable environment for evolution of bacteria towards broad phage resistance although this was not observed experimentally either here or by others [19].

We showed that, in the tested strains, the presence of a CRISPR-Cas system was not associated with resistance to a particular lytic phage. On the contrary, it appears that a CRISPR-Cas system was absent from the strains that could not support multiplication of any tested phages. However, in the light of the recent discovery in some Mu-like phages of genes that inactivate the CRISPR-Cas systems it is not possible to completely rule out a role of this system in controlling lytic phage infection [26]. None of the phages present in this study belong to the Mu-like genus and we did not find a candidate gene in the configuration described by Bondy-Denomy et al. in the two sequenced genomes. Cady et al. analysed the prevalence of the two CRISPR-Cas systems in *P. aeruginosa* and showed that the 132 viral spacers with matches in sequence databases matched temperate bacteriophage/prophages capable of inserting into the host chromosome; none matched extrachromosomally replicating lytic *P. aeruginosa* bacteriophages [39]. They later reported that the type I–F (*Yersinia*) CRISPR region of *P. aeruginosa* strain UCBPP-PA14 plays a role in immunity against specific temperate phages [24]. In the strains used here, the majority of spacers with homologues in databases also match temperate phages. We observed some matches with chromosomal sequences such as pyocins and genes of pathogenicity islands, both of which often appear to have their origins in temperate phages. The CRISPR-Cas system might thus be used to inhibit reactivation of prophages or transposable sequences. We confirmed that the majority of clinical *P. aeruginosa* strains are lysogenic (often multilysogenic), and that spontaneous activation of these phages is frequent, as previously reported [25], 61. In *E. coli* and in *Streptococcus pyogenes* the CRISPR-Cas system is also involved in the control of lysogenisation and prophage induction [62]. To understand the role of the CRISPR-Cas system it will be necessary to further explore its function during the establishment and control of lysogeny.

## Conclusion

Our results emphasize the difficulty in preparing a phage cocktail that could be efficient on the whole species, particularly given the wide diversity of the *P. aeruginosa* species. Several bacterial strains cannot support growth of all lytic phages investigated in this study and phages from other collections, representing a total of 6 different genera, which suggests that they may have developed new resistance mechanisms. These strains will need to be analysed in detail and their genomes fully sequenced to try and identify the involved mechanisms, including possible prophage-based ones, and look for ways of bypassing them.

## Supporting Information

Figure S1 **Minimum spanning tree representation of the clustering of 325** ***P. aeruginosa*** **strains from CF patients.** The 50 strains selected for phage isolation are colored in red.
(PPT)

Figure S2 **Restriction pattern of 5 pyophage-derived phages using** ***Eco*** **RI and** ***Hind*** **III.** The fragment sizes were measured using the BioNumerics software. Lanes 1 to 6, *Hind*III-digested lambda DNA used as size marker, P1-15_pyo_, P8-13_pyo_, P2-10_pyo_, P3-20_pyo_, PTr60_pyo_. Double or triple bands were evaluated by analyzing the band intensity.
(PPT)

Figure S3 **Distribution of nicks along the genome of phage P1-14_Or01_.** A) Sequence read coverage as observed in BioNumerics. B) Alignment of sequences at the nick site. The consensus motif is shown in red. The <> symbol in the center of the motif indicates the end of all the sequencing reads.
(PPT)

Figure S4 **Analysis of the** ***Yersinia*** **CRISPR-Cas system.** PCR amplification of A) CRISPR1, B) CRISPR2 and C) *cas*1 in 19 selected strains and reference strain PA14.
(PPT)

Figure S5 **Schematic representation of CRISPR1 spacer organization in clusters of strains.** The CRISPR is oriented with the leader on the left, corresponding to the growing end where the more recently added spacers are found. The alignment is the output of a CRISPRtionary analysis after the Re-annote Spacers function has been activated.
(DOC)

Figure S6 **Comparison of MLVA clustering and CRISPR 1 content.** On the left is shown a dendrogram produced from MLVA data. Colors indicate strains whose CRISPR1 possesses common spacers. On the right are shown the spacer organization in the four larger clusters.
(PPT)

Table S1 **Sequence of 264 CRISPR1 spacers and distribution into the 42 tested strains.**
(XLS)

Table S2 **Combined list of spacers from Cady et al.** [**39**] **and from the present study.**
(XLS)
