# Supplementary figures and images for: The Susceptibility of Pseudomonas aeruginosa Strains from Cystic Fibrosis Patients to Bacteriophages
Source: PLoS One. 2013 Apr 24;8(4):e60575. doi: 10.1371/journal.pone.0060575 (PMC3634792; doi:10.1371/journal.pone.0060575)

## Slide 1
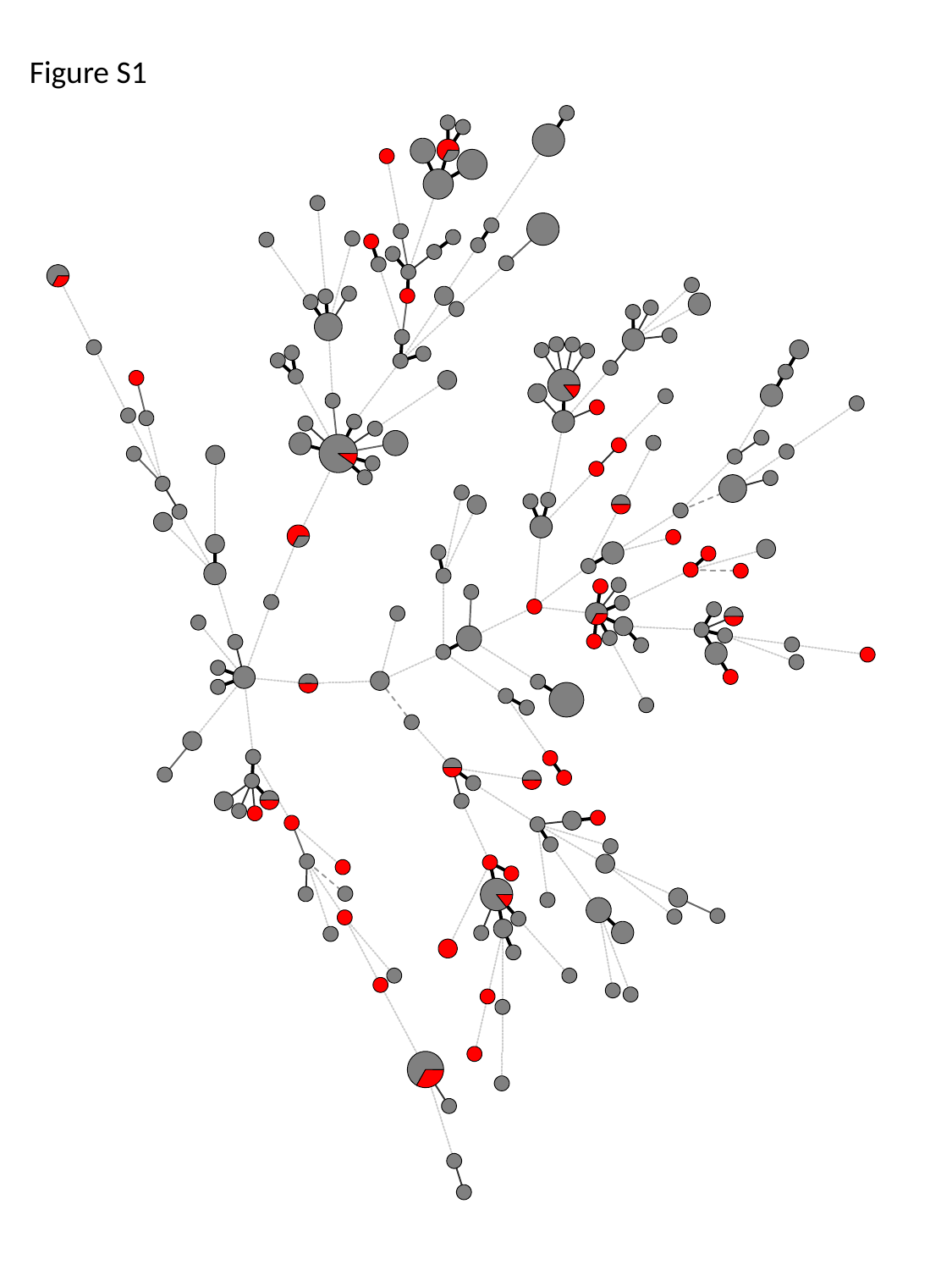

Figure S1

Supplement: Figure S1 — Minimum spanning tree representation of the clustering of 325 P. aeruginosa strains from CF patients. The 50 strains selected for phage isolation are colored in red. (PPT) [file pone.0060575.s001.ppt]

## Slide 1
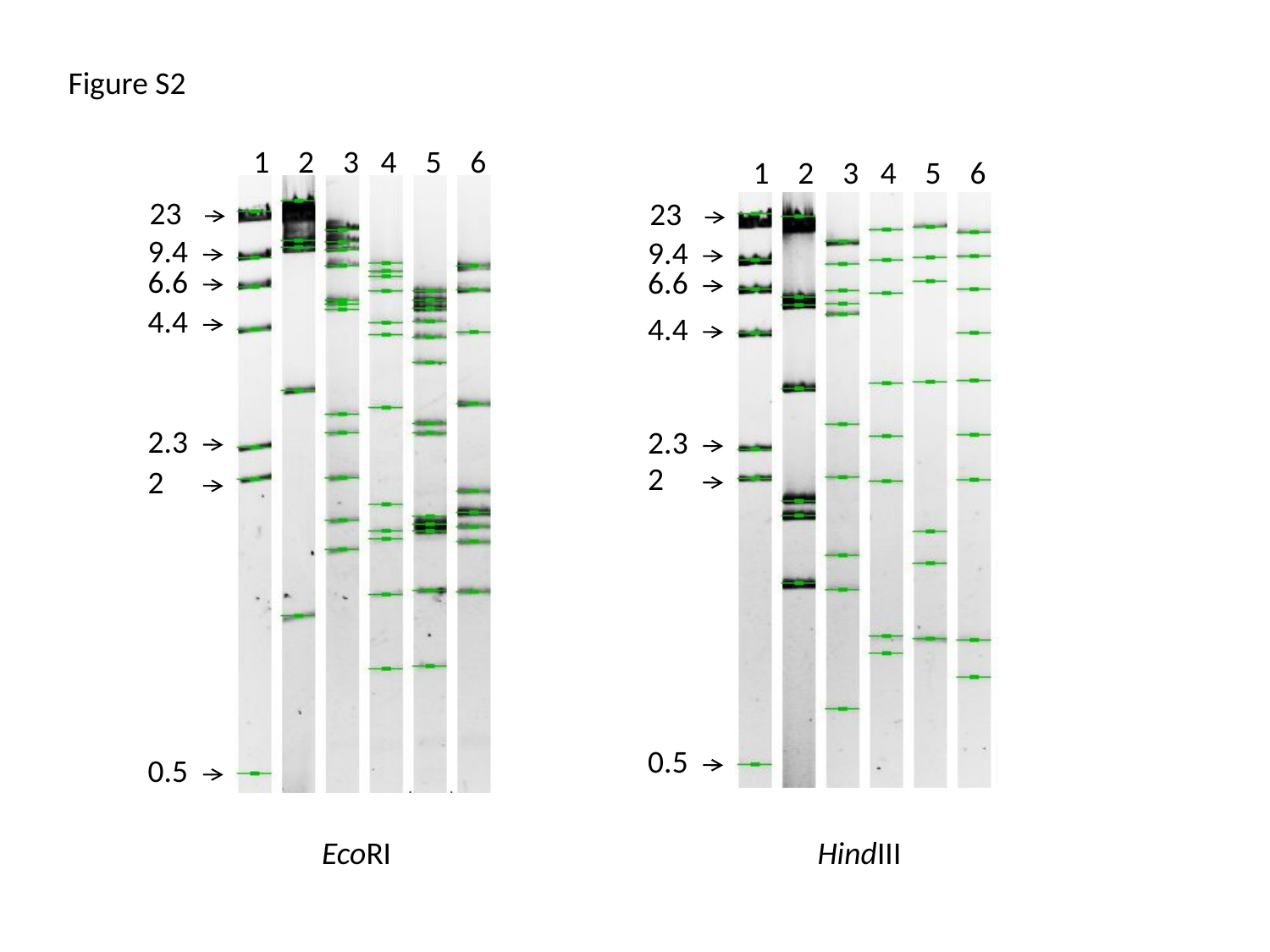

Figure S2
1 2 3 4 5 6
1 2 3 4 5 6
23
23
9.4
9.4
6.6
6.6
4.4
4.4
2.3
2.3
2
2
0.5
0.5
EcoRI
HindIII

Supplement: Figure S2 — Restriction pattern of 5 pyophage-derived phages using Eco RI and Hind III. The fragment sizes were measured using the BioNumerics software. Lanes 1 to 6, HindIII-digested lambda DNA used as size marker, P1-15pyo, P8-13pyo, P2-10pyo, P3-20pyo, PTr60pyo. Double or triple bands were evaluated by analyzing the band intensity. (PPT) [file pone.0060575.s002.ppt]

## Slide 1
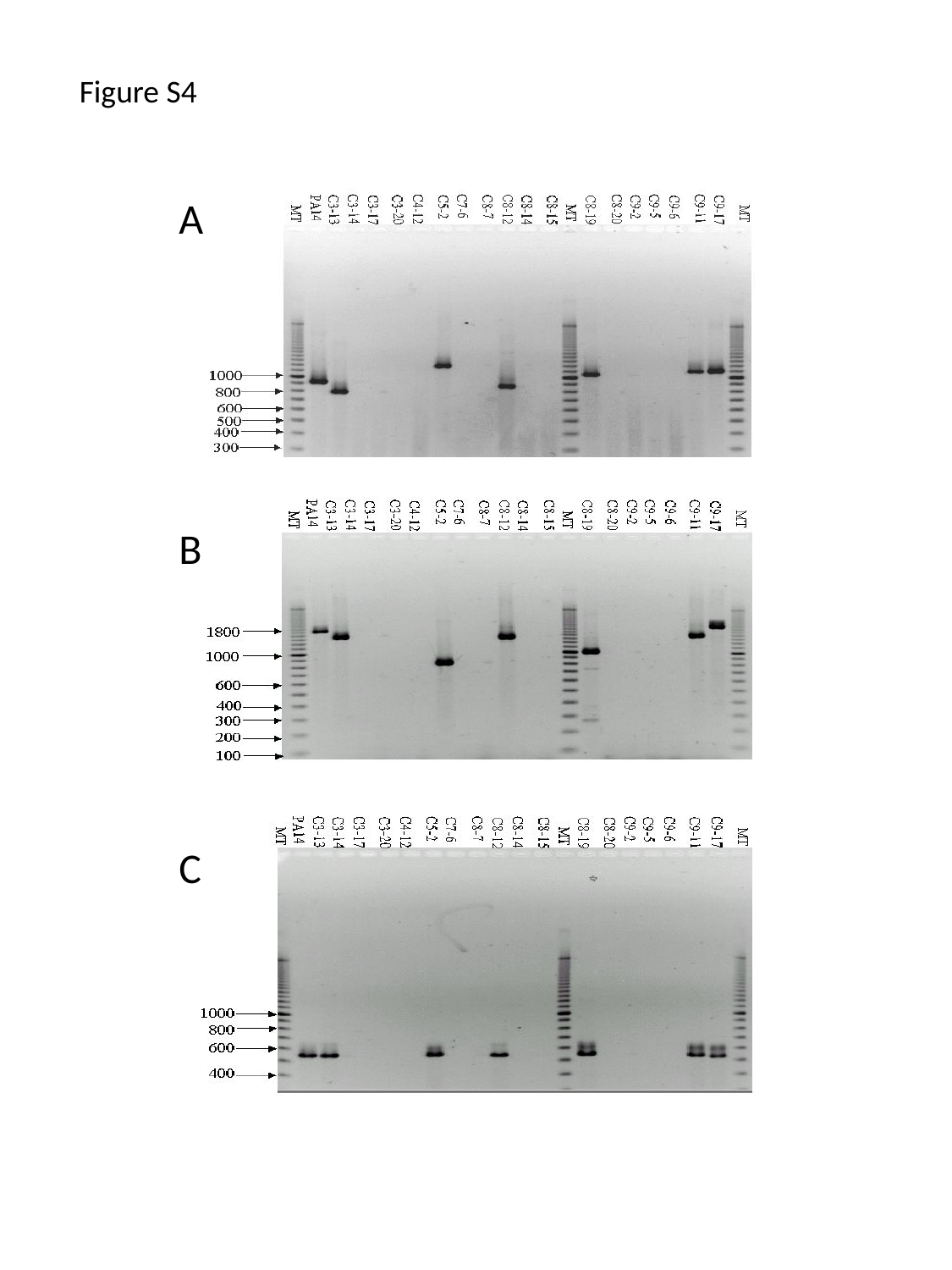

Figure S4
A
B
C

Supplement: Figure S4 — Analysis of the Yersinia CRISPR-Cas system. PCR amplification of A) CRISPR1, B) CRISPR2 and C) cas1 in 19 selected strains and reference strain PA14. (PPT) [file pone.0060575.s004.ppt]

Cluster PA14


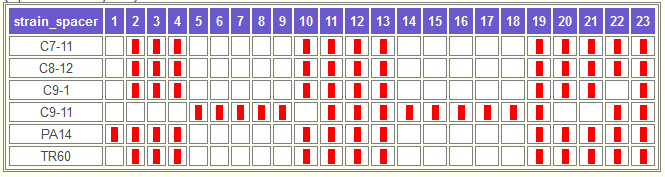


Cluster C50


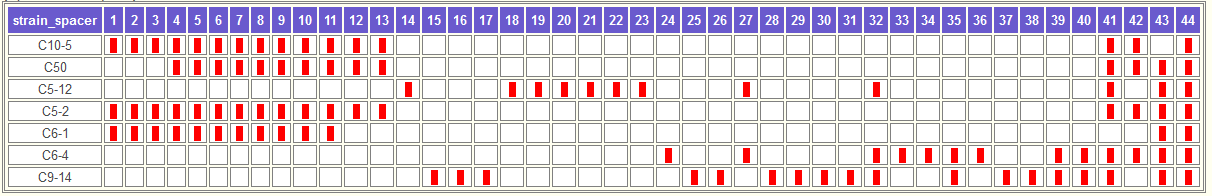


Other clusters


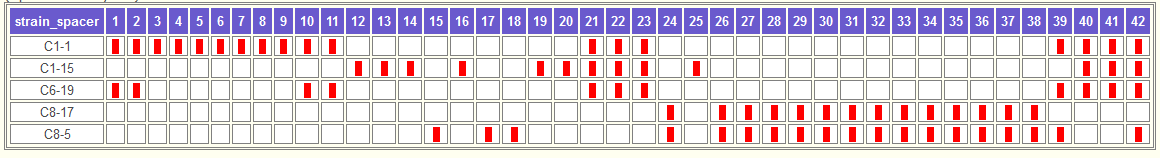


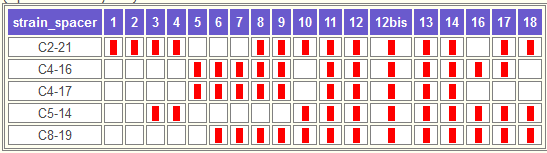


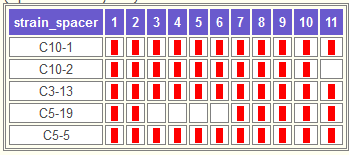


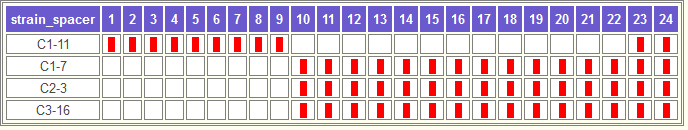


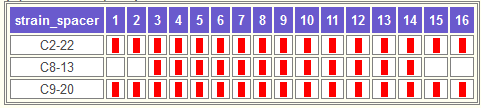


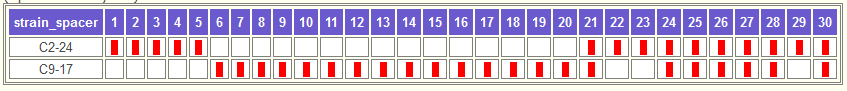

Supplement: Figure S5 — Schematic representation of CRISPR1 spacer organization in clusters of strains. The CRISPR is oriented with the leader on the left, corresponding to the growing end where the more recently added spacers are found. The alignment is the output of a CRISPRtionary analysis after the Re-annote Spacers function has been activated. (DOC) [file pone.0060575.s005.doc]

## Slide 1
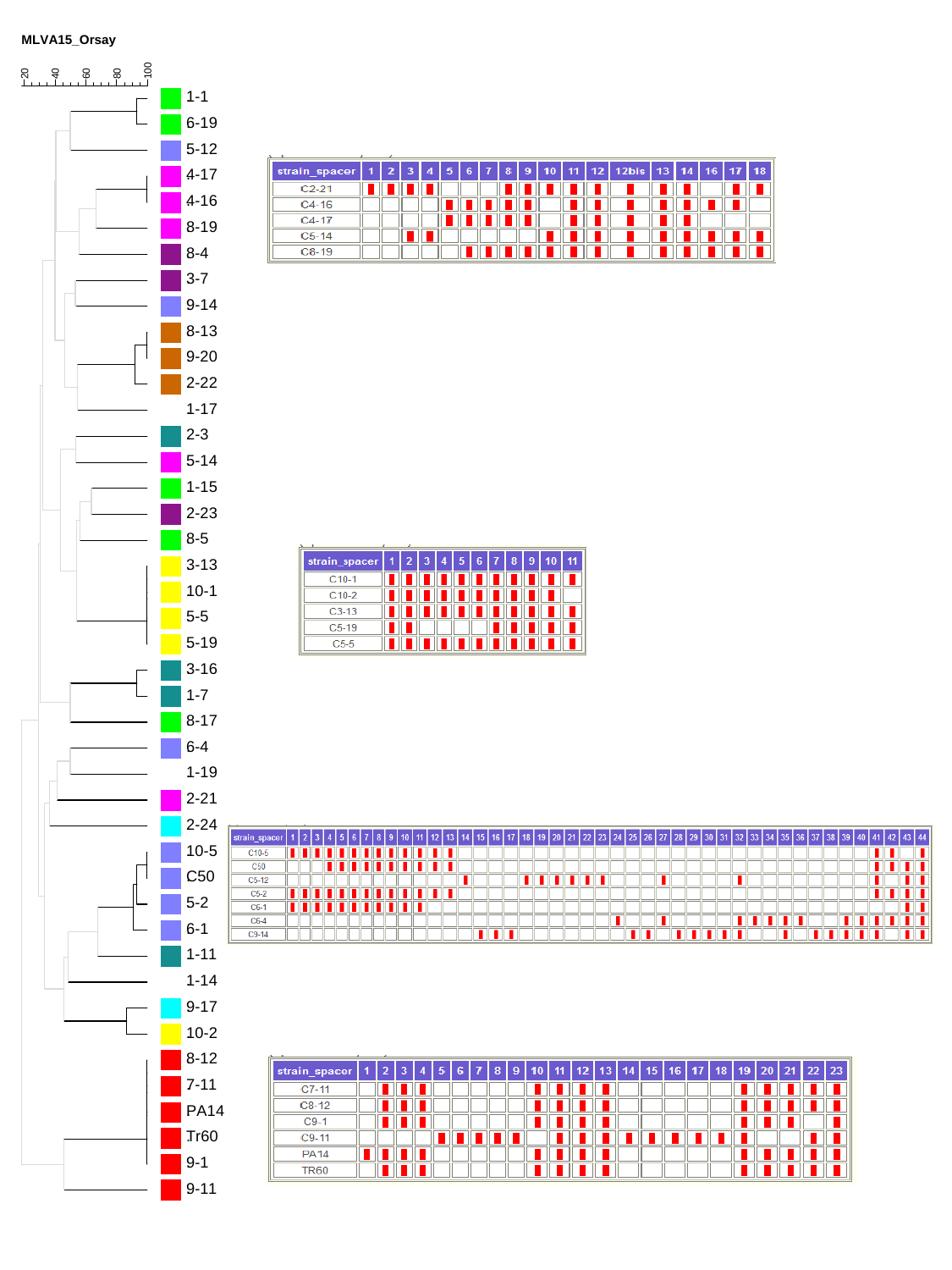

Supplement: Figure S6 — Comparison of MLVA clustering and CRISPR 1 content. On the left is shown a dendrogram produced from MLVA data. Colors indicate strains whose CRISPR1 possesses common spacers. On the right are shown the spacer organization in the four larger clusters. (PPT) [file pone.0060575.s006.ppt]
