# Supplementary material for: The Susceptibility of Pseudomonas aeruginosa Strains from Cystic Fibrosis Patients to Bacteriophages
Source: PLoS One. 2013 Apr 24;8(4):e60575. doi: 10.1371/journal.pone.0060575 (PMC3634792; doi:10.1371/journal.pone.0060575)
Supplement: Figure S3 — Distribution of nicks along the genome of phage P1-14Or01. A) Sequence read coverage as observed in BioNumerics. B) Alignment of sequences at the nick site. The consensus motif is shown in red. The <> symbol in the center of the motif indicates the end of all the sequencing reads. (PPT) [file pone.0060575.s003.ppt]

## Slide 1
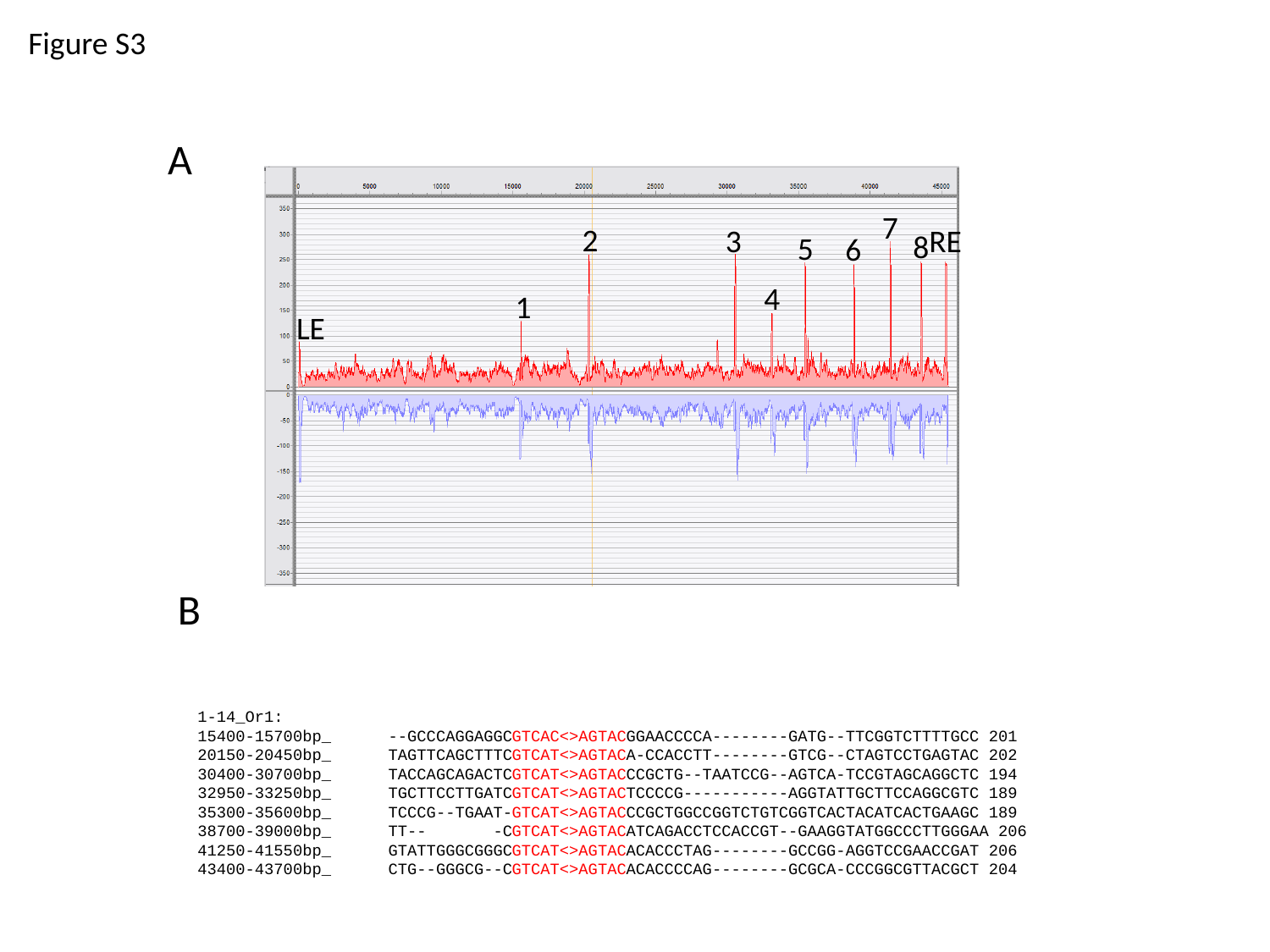

Figure S3
A
7
2
3
RE
8
5
6
4
1
LE
B
1-14_Or1:
15400-15700bp_ --GCCCAGGAGGCGTCAC<>AGTACGGAACCCCA--------GATG--TTCGGTCTTTTGCC 201
20150-20450bp_ TAGTTCAGCTTTCGTCAT<>AGTACA-CCACCTT--------GTCG--CTAGTCCTGAGTAC 202
30400-30700bp_ TACCAGCAGACTCGTCAT<>AGTACCCGCTG--TAATCCG--AGTCA-TCCGTAGCAGGCTC 194
32950-33250bp_ TGCTTCCTTGATCGTCAT<>AGTACTCCCCG-----------AGGTATTGCTTCCAGGCGTC 189
35300-35600bp_ TCCCG--TGAAT-GTCAT<>AGTACCCGCTGGCCGGTCTGTCGGTCACTACATCACTGAAGC 189
38700-39000bp_ TT-- -CGTCAT<>AGTACATCAGACCTCCACCGT--GAAGGTATGGCCCTTGGGAA 206
41250-41550bp_ GTATTGGGCGGGCGTCAT<>AGTACACACCCTAG--------GCCGG-AGGTCCGAACCGAT 206
43400-43700bp_ CTG--GGGCG--CGTCAT<>AGTACACACCCCAG--------GCGCA-CCCGGCGTTACGCT 204
